# Induction of thymidine phosphorylase as a pharmacodynamic end-point in patients with advanced carcinoma treated with 5-fluorouracil, folinic acid and interferon alpha

**DOI:** 10.1054/bjoc.2000.1230

**Published:** 2000-06-15

**Authors:** J P Braybrooke, D J Propper, K J O’Byrne, M I Koukourakis, A V Patterson, S Houlbrook, S D Love, S Varcoe, M Taylor, T S Ganesan, D C Talbot, A L Harris

**Affiliations:** ICRF Medical Oncology Unit, Churchill Hospital, Oxford, OX3 7LJ, UK; Department of Radiotherapy and Oncology, University Hospital of Iraklion, Iraklion, Crete, 71110, Greece

**Keywords:** thymidine phosphorylase, interferon alpha, 5-fluorouracil, folinic acid

## Abstract

Thymidine phosphorylase (TP) is an essential enzyme for the biochemical activation of 5-fluorouracil (5-FU). Interferon upregulates TP in vivo, although the dose and schedule of interferon for optimal biomodulation of 5-FU is not known. In this study, TP activity was measured in peripheral blood lymphocytes (PBLs) from patients with advanced carcinoma receiving treatment with 5-FU and folinic acid. Cohorts of patients were treated with interferon alpha (IFNα), immediately prior to 5-FU/folinic acid, at doses of 3 MIU m^–2^, 9 MIU m^–2^and 18 MIUm^–2^. IFNα was administered on day 0 cycle two, day –1 and day 0 cycle three and day –2, day –1 and day 0 cycle four. A fourth cohort was treated with IFNα 9 MIU m^–2^three times per week from cycle 2 onwards. Twenty-one patients were entered into the study with 19 evaluable for response. Six patients (32%) had stable disease and 13 (68%) progressive disease. There were no grade-IV toxicities. TP activity was detected in PBLs from all patients with wide interpatient variability in constitutive TP activity prior to chemotherapy, and in response to IFNα. 5-FU/folinic acid alone did not induce TP activity but a single dose of IFNα led to upregulation of TP within 2 h of administration with a further increase by 24 h (signed rank test, *P* = 0.006). TP activity remained elevated for at least 13 days (signed rank test, *P* = 0.02). There were no significant differences in TP activity between schedules or with additional doses of IFNα. A single dose of IFNα as low as 3 MIU m^–2^can cause sustained elevation of PBL TP activity in vivo indicating that biochemical markers are important pharmacodynamic endpoints for developing optimal schedules of IFNα for biomodulation of 5-FU. © 2000 Cancer Research Campaign


British Journal of Cancer (2000) **83**(2), 219–224
© 2000 Cancer Research Campaign
doi: 10.1054/ bjoc.2000.1230, available online at http://www.idealibrary.com on IDEAL®

### Induction of thymidine phosphorylase as a pharmacodynamic end-point in patients with advanced carcinoma treated with 5-fluorouracil, folinic acid and interferon alpha

**JP Braybrooke[1], DJ Propper[1], KJ O'Byrne[1], MI Koukourakis[2], AV Patterson[1], S Houlbrook[1], SD Love[1], S Varcoe[1], M Taylor[1], TS Ganesan[1], DC Talbot[1] and AL Harris[1]**

[1]ICRF Medical Oncology Unit, Churchill Hospital, Oxford, OX3 7LJ, UK; [2]Department of Radiotherapy and Oncology, University Hospital of Iraklion, Iraklion 71110, Crete, Greece

**Summary** Thymidine phosphorylase (TP) is an essential enzyme for the biochemical activation of 5-fluorouracil (5-FU). Interferon upregulates TP in vivo, although the dose and schedule of interferon for optimal biomodulation of 5-FU is not known. In this study, TP activity was measured in peripheral blood lymphocytes (PBLs) from patients with advanced carcinoma receiving treatment with 5-FU and folinic acid. Cohorts of patients were treated with interferon alpha (IFNα), immediately prior to 5-FU/folinic acid, at doses of 3 MIU $m^{-2}$, 9 MIU $m^{-2}$ and 18 MIU$m^{-2}$. IFNα was administered on day 0 cycle two, day –1 and day 0 cycle three and day –2, day –1 and day 0 cycle four. A fourth cohort was treated with IFNα 9 MIU $m^{-2}$ three times per week from cycle 2 onwards. Twenty-one patients were entered into the study with 19 evaluable for response. Six patients (32%) had stable disease and 13 (68%) progressive disease. There were no grade-IV toxicities. TP activity was detected in PBLs from all patients with wide interpatient variability in constitutive TP activity prior to chemotherapy, and in response to IFNα. 5-FU/folinic acid alone did not induce TP activity but a single dose of IFNα led to upregulation of TP within 2 h of administration with a further increase by 24 h (signed rank test, $P = 0.006$). TP activity remained elevated for at least 13 days (signed rank test, $P = 0.02$). There were no significant differences in TP activity between schedules or with additional doses of IFNα. A single dose of IFNα as low as 3 MIU $m^{-2}$ can cause sustained elevation of PBL TP activity in vivo indicating that biochemical markers are important pharmacodynamic endpoints for developing optimal schedules of IFNα for biomodulation of 5-FU. © 2000 Cancer Research Campaign

**Key words**: thymidine phosphorylase; interferon alpha; 5-fluorouracil; folinic acid

Thymidine phosphorylase (TP) catalyses the reversible synthesis of thymidine, and inorganic phosphate, from thymine using deoxyribose-1-phosphate as a co-substrate. TP is present in a wide range of normal human tissues (Fox et al, 1995), with elevated expression in many tumours including breast, lung, bladder, colorectal, and gastric cancers (Fox et al, 1996; Giatromanolaki et al, 1997; O'Brien et al, 1996; Takahashi et al, 1998; Takebayashi et al, 1996; Yoshikawa et al, 1999). TP is important in tumour angiogenesis (Moghaddam and Bicknell, 1992; Moghaddam et al, 1995) and elevated levels in cancer cells can be exploited in cytotoxic chemotherapy for the activation of the fluoropyrimidine, 5-fluorouracil (5-FU).

TP converts 5-FU to 2′-deoxy-5-fluorouridine (FUdR) which in turn is metabolized by thymidine kinase (TK) to 5-fluoro-deoxyuridine monophosphate (FdUMP). FdUMP forms a ternary complex with 5,10 methylene-tetrahydrofolate (5,10-$CH_2$-THF) and thymidylate synthase (TS) leading to defective DNA synthesis and repair. Other biochemical pathways involving TS include metabolism of 5-FU to 5-fluorouridine diphosphate (FUDP), which interferes with glycosylation of proteins and lipids, and 5-fluorouridine triphosphate (FUTP) which is incorporated into RNA. Attempts to further enhance the cytotoxicity of 5-FU in clinical trials have developed from in vitro models that demonstrate enhanced activity following biochemical modulation with agents such as folinic acid (calcium folinate) and interferon (Moran and Scanlon, 1991; Wadler et al, 1990*b*).

The addition of folinic acid to 5-FU-containing schedules enhances the binding of FdUMP to the ternary complex leading to greater cytotoxicity. Similarly, in vitro interferon α (IFNα) increases TP levels with a corresponding increase in FdUMP formation, and causes single- and double-strand DNA breaks in colon carcinoma cell lines (Houghton et al, 1993; Schwartz et al, 1994; 1995; Wadler et al, 1990*b*, 1996). Modulation of 5-FU metabolism by folinic acid or IFNα alone has been assessed in a number of clinical trials in patients with colorectal cancer with evidence for enhanced clinical response (ACCM-AP, 1992; Corfu-A Study Group, 1995; Ragnhammar et al, 1995; Wadler et al, 1991). However, double modulation of 5-FU with folinic acid and IFNα has not enhanced efficacy compared to 5-FU and folinic acid alone, but has increased toxicity (Kosmidis et al, 1996; Seymour et al, 1996; Woolmark et al, 1998). A wide range of schedules have been studied, but most trials have not used biochemical or pharmacological markers to assess the optimal combination in terms of dose and frequency of administration of

*Received 6 October 1999*
*Revised 2 March 2000*
*Accepted 10 March 2000*

*Correspondence to:* AL Harris

IFNα in conjunction with 5-FU and folinic acid (Wadler et al, 1990*a*).

IFNα does not directly modify 5-FU pharmacokinetics in vivo (Seymour et al, 1994*b*) but induces TP both in vitro (Schwartz et al, 1995) and in vivo. Makower et al (1997) measured TP mRNA and protein levels in peripheral blood lymphocytes from patients with advanced malignancy receiving treatment with 5-FU and either IFNα or β at doses of 5–10 MIU. Fifteen of 21 (71%) patients exhibited an increase in TP protein levels of two-fold or greater after a single dose of IFNα or β, with elevated levels persisting for up to 48 h. There was a corresponding rise in TP mRNA levels in these patients demonstrating that one of the actions of interferon is to induce TP by regulation of the level of mRNA expression.

The effect of different doses and schedules of IFNα on TP induction and the subsequent effect on both 5-FU activation and tumour angiogenesis in vivo is not known. In this study, in order to try and develop a rational basis for 5-FU modulation, we assess the effect of double biomodulation of a standard schedule of 5-FU with folinic acid and IFNα on peripheral blood lymphocyte TP activity. The dose of interferon, and schedule of administration, was escalated during the study in order to evaluate which dose and schedule of IFNα gives the highest TP induction for longest. We demonstrate that 5-FU and folinic acid alone do not increase TP protein activity but that a single dose of IFNα as low as 3 MIU $m^{-2}$ rapidly induces TP and that this can remain elevated for up to 13 days.

### PATIENTS AND METHODS

### Patients

Patients with progressive advanced carcinoma suitable for treatment with 5-FU/folinic acid or those with carcinoma refractory to conventional treatment schedules were entered into the study. Eligibility criteria included histological or cytological proof of diagnosis, age over 18 years, written informed consent, measurable or evaluable disease and adequate bone marrow, renal and hepatic function. Patients were excluded if they had received chemotherapy within the past 4 weeks, had concurrent infection or a history of autoimmune disease or major psychiatric disorder. All patients gave written informed consent and the trial was conducted with the approval of the Central Oxford Research Ethics Committee.

### Drug administration

5-FU and folinic acid were administered every 2 weeks in hospital using a modified De-Gramont schedule (De Gramont et al, 1988) to a maximum of eight cycles. Folinic acid 200 mg $m^{-2}$ in 250 ml 0.9% saline was given as an i.v. infusion over 2 h on day 0, immediately followed by 5-FU 400 mg $m^{-2}$ in 100 ml 0.9% saline i.v. over 10 min with a subsequent continuous i.v. infusion of 5-FU 400 mg $m^{-2}$ in 1l 0.9% saline over 22 h. This was repeated on day 1. IFNα (Roferon-A, Roche, Welwyn Garden City, UK) was administered s.c. prior to each administration of 5FU/folinic acid on cycle two and subsequent cycles. There was a within-patient and between-patient dose escalation of IFNα. Patients received no IFNα on cycle one, treatment with IFNα on day 0 of cycle two, day –1 and day 0 of cycle three and day –2, day –1 and day 0 of cycle four. Cohort one was treated with IFNα 3 million international units (MIU) $m^{-2}$ at each dose, cohort two with 9 MIU $m^{-2}$ and cohort three with 18 MIU $m^{-2}$. Cohort four received a more conventional schedule of IFNα administered at 9 MIU $m^{-2}$ on day 0, 2 and 4 during the week that they were being treated with 5FU/folinic acid. This schedule was given from cycle two of treatment with no further escalation in dose for cycles three and four. IFNα, where possible, was administered at the same time each day to minimize the possibility of error caused by circadian variability in TP activity.

Antiemetic prophylaxis consisted of metoclopramide 10 mg q.d.s/p.r.n. Patients who experienced fever following IFNα were treated with paracetamol 1 g q.d.s. and, if fever remained uncontrolled naproxen 500 mg b.d. This was continued as prophylaxis in these patients prior to further interferon. All other concurrent medication was permitted with the exception of steroids unless the patient remained on a stable dose throughout the cycles of treatment.

Treatment was delayed for 1 week in patients with absolute neutrophil count < $1.5 \times 10^9$ $l^{-1}$ or platelet count < $100 \times 10^9$ $l^{-1}$. Treatment was also delayed for any grade III/IV non-haematological toxicity until that toxicity had resolved to grade I or less. The dose of IFNα was reduced to the treatment level in the previous cohort in patients who did not tolerate the prescribed dose or whose treatment was delayed for more than 1 week. Toxicity was graded according to WHO criteria and disease response was evaluated clinically and by CT scan after four courses and at the completion of treatment. Standard WHO criteria for objective tumour response assessment were employed.

### Blood samples

Venous blood samples were taken during the first four cycles of the study for lymphocyte separation in order to monitor the effect of treatment on TP levels in peripheral blood lymphocytes (PBL). For cohorts one, two and three, on cycle one, blood was taken prior to treatment (day 0) and on days 1, 2 and 7. For subsequent cycles samples were taken prior to each dose of IFNα and on days 0, 1, 2 and 7. After the first dose of IFNα on cycle two only, an additional sample was taken at +2 h. For cohort four, on the conventional schedule of IFNα, blood was taken on days 0, 2 and 7 only.

10 ml venous blood samples were collected at each time point into glass ‘vacutainer’ tubes (Becton Dickinson, New York, USA) containing EDTA and placed on ice before processing. Lymphocytes were separated over ‘lymphoprep’ (Nicomed, Majorstua, Norway) under aseptic conditions and centrifuged at 1500 ***g*** for 20 min. The plasma supernatant was aspirated and the lymphocyte pellet was frozen at –70°C until analysis.

### Biochemical evaluation of thymidine phosphorylase

Lymphocyte pellets were thawed, washed and re-suspended in 1 ml Tris-HCl buffer (50 mM Tris-CL, 150 mM NaCl, 0.05 mM DTT, 0.05 mM PMSF, pH 7.4) at 4°C. Cells were sonicated on ice before centrifuging at 10 000 ***g*** for 30 min. The supernatant was harvested and stored in liquid nitrogen until analysis. Total protein content for each of the cell lysates was determined by the Bradford assay (Bio-Rad, Hemel Hempstead, UK) and quantified using a BSA standard (Sigma, Poole, UK, 1 mg $ml^{-1}$).

Thymidine phosphorylase activity assays were performed by spectrophotometric monitoring and have been described previously (Patterson et al, 1995). Briefly, 20 µg of lymphocyte

© 2000 Cancer Research Campaign cytosolic protein was incubated with 10 mM thymidine, 50 mM$K_3PO_4$, pH 7.4 in a final volume of 300 μl for 16 h at 37°C. The reaction was terminated by the addition of 700 μl of ice-cold 0.5 M NaOH to produce a final solution pH of 13.3. The formation of thymine from thymidine was determined by measuring absorbance in a spectrophotometer at 300 nm. Total thymine formation was quantified against a known standard (range 0.1–1 mM) and activity expressed as nmol thymine released $hr^{-1}$ $mg^{-1}$ protein. There was no significant difference between repeat assays of the same sample (paired *t* = test, *P* = 0.9, *n* = 15).

### Correlation of TP protein levels with TP protein activity

PBLs collected from five patients in the study were assayed for TP activity as described above and for total TP protein levels determined by protein immunoblots using the anti-TP antibody PG44c (kindly supplied by Dr R Bicknell, ICRF) (Moghaddam et al, 1995). Total TP protein levels at each time-point were assessed by measuring absorbance on the Bio-imager (Milligen/Biosearch, Millipore). There was a strong positive correlation between the two techniques (Spearmans correlation coefficient, *rho* = 0.83, *P* < 0.001) and all other samples were therefore only analysed for TP activity.

### Statistical methods

Using the method of summary measures (Matthews et al, 1990), the area under the TP curve (AUC), adjusting for baseline TP (where adjustment is value minus single pretreatment TP value), was chosen for analysis in order to evaluate which dose (cohort) and which schedule (cycle) of IFNα gives the highest TP induction for longest. Since the data did not differ appreciably from independence, ANOVA was the final method used to predict the optimal dose and schedule of IFNα for TP induction.

Initially, to assess the effect of 5FU/folinic acid alone, a Wilcoxon signed rank test was used to compare matched pairs of TP values on day 0 with days 1, 2, 7 and 13 of cycle one, including patients from all four cohorts. To consider the effect of a single dose of interferon on TP levels signed rank tests were used for paired values for cycle two, cohorts one–three, for day 0 and data from day 0 + 2 h, day 1, day 2, day 7 and day –1 of cycle three. The question of whether interferon had a carryover effect on baseline TP levels between cycles was compared using Friedman's non parametric two-way analysis of variance.

When using the raw TP values, non-parametric tests were necessary because the data did not show a normal distribution.

### RESULTS

Twenty-one patients were entered into the study (median age 58 years, range 46–72). Sites of primary tumour included renal (five patients), colorectal (three), breast (three), unknown primary adenocarcinoma (three), pancreas (two), gastric, bladder, cholangiocarcinoma, carcinoid and lung adenocarcinoma (one each). Seven patients were previously untreated with chemotherapy and ten patients had received one prior chemotherapy schedule, of whom two had received 5-FU. Four patients had previously received two chemotherapy schedules.

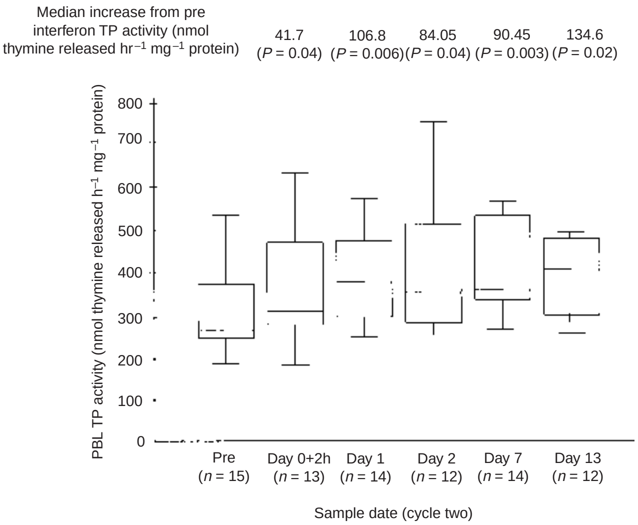

**Figure 1** Box whisker plot showing median induction of PBL TP activity in cycle two following a single dose of IFNα for cohorts one–three. PBLs were collected prior to IFNα on day 0 and then at day 0 + 2 h, day 1, day 2, day 7, and day 13. 5-FU/folinic acid was commenced immediately after the IFNα on day 0. The 'box' extends to the interquartile range and the 'whiskers' to 1.5 times the interquartile range. There was a significant induction of TP activity, compared to cycle two day 0, within 2 h of IFNα with a further increase by 24 h. TP activity remained elevated for at least 13 days.

### Thymidine phosphorylase activity

TP activity was detected in PBLs in all patients with wide interpatient variability in the constitutive level prior to any chemotherapy (median = 329.2 nmol thymine released $hr^{-1}$ $mg^{-1}$ protein, range 187–587, *n* = 19). There was no significant increase in TP activity during cycle one when patients were treated with 5-FU/folinic acid alone (signed rank test, *P* > 0.05 in all cases, *n* = 19).

### Immediate effect of IFNα

The effect of the first dose of IFNα on lymphocyte TP activity in patients from cohorts one to three was evaluated in cycle two by paired analysis of pre-IFNα samples with those taken at day 0 + 2 h, day 1, day 2, day 7 and day 13. An immediate effect of IFNα on TP levels was observed at 2 h (median increase = 41.7 nmol thymine released $h^{-1}$ $mg^{-1}$ protein, signed rank test, *P* = 0.04, *n* = 13), with a further increase in TP activity by 24 h (median increase = 106.8 nmol thymine released $h^{-1}$ $mg^{-1}$ protein, signed rank test, *P* = 0.006, *n* = 14). TP activity remained elevated compared to baseline at day 7 of cycle two (median increase = 90.45 nmol thymine released $h^{-1}$ $mg^{-1}$ protein, signed rank test, *P* = 0.003, *n* = 14) with persisting upregulation at day 13 (median increase 134.6 nmol thymine released $h^{-1}$ $mg^{-1}$ protein, signed rank test, *P* = 0.02; *n* = 12) (Figure 1). There was considerable variation between patients in the response of their PBL TP activity to the first dose of IFNα. Nine out of 14 patients (64%) sampled 24 h after the first dose of IFNα, had a greater than 20% increase in TP activity with three (22%) showing no change and two (14%) a decrease. Of the nine patients whose TP activity was elevated 24 h after a single dose of IFNα, it remained elevated for up to eight days in seven patients (78%) and was still greater than 20% above baseline in five out of eight (62.5%) of those patients assessed at day 13. Figure 2 shows the variation in PBL TP activity in one

© 2000 Cancer Research Campaign

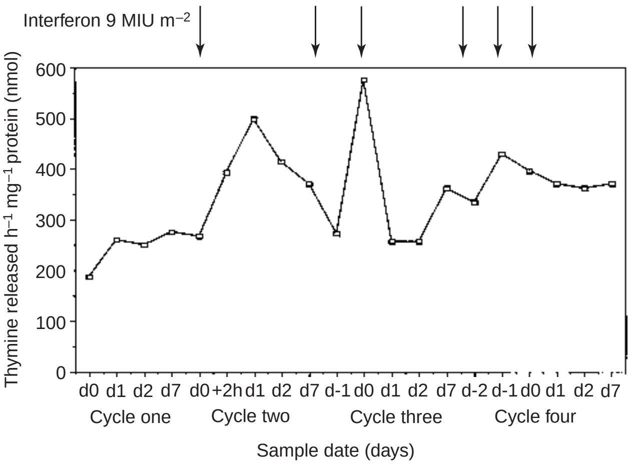

**Figure 2** Change in PBL TP activity in a single patient treated with a standard schedule of 5-FU/folinic acid and IFNα 9 MIU $m^{-2}$ from cycle two onwards. The days of administration of IFNα are indicated by arrows. Data for PBL TP activity is expressed as thymine released $h^{-1}$ $mg^{-1}$ protein (nmol) (Y-axis). X-axis = sample date and cycle.

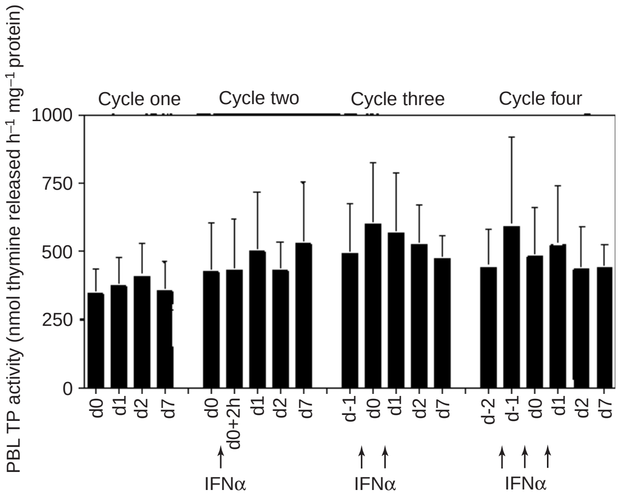

**Figure 3** Effect of IFNα on PBL TP activity (all cohorts). IFNα was administered on day 0 cycle two, day –1, day 0 cycle three and day –2, day –1, day 0 cycle four) cohorts one–three. Cohort four received IFNα days 0, 2 and 4 from cycle two onwards. PBL TP activity, expressed as thymine released $h^{-1}$ $mg^{-1}$ protein (nmol), is pooled for all cohorts and expressed as a mean for all samples (Y-axis). The days of administration of IFNα are indicated by arrows. Bars = SEM. X-axis = sample date and cycle.

patient who demonstrated an increase in TP activity with doses of IFNα.

### Duration of effect of IFNα

The prolonged effects of a single dose of IFNα on TP activity for the 14 days between chemotherapy cycles was evaluated by comparing the baseline TP activity prior to cycle two with baseline TP activity in cycle three and cycle four (Figure 3). In cohorts one to three, analysed together, there was an increase in baseline TP activity prior to cycle three (median = 415.3 nmol thymine released $h^{-1}$ $mg^{-1}$ protein, range 272–1069, Friedman's non parametric, $P = 0.009$; $n = 14$). There was no further increase in baseline TP activity at cycle four (median = 349.6 nmol thymine released $h^{-1}$ $mg^{-1}$ protein, range 296–806, $n = 14$).

### Dose response of IFNα

One of the aims of this study was to assess which of the four schedules of IFNα used could be recommended for larger studies based on biochemical analysis of TP activity. The AUC of TP activity, adjusting for baseline, did not show a statistically significant difference between doses or between cycles (ANOVA, $P > 0.1$ in all cases). Similarly there was no significant difference in TP induction by the same total dose of IFNα administered by different schedules, e.g. 9 MIU/$m^2$ as a single dose compared with $3 \times 3$ MIU $m^{-2}$ (ANOVA, $P = 0.9$).

### Toxicity

A total of 85 cycles of chemotherapy were administered with a median of four per patient. Two patients received the maximum of eight cycles and two discontinued after only one cycle. The main toxicities observed were diarrhoea (WHO grade 2 or 3, $n = 5$), nausea and vomiting ($n = 6$), pyrexia ($n = 12$), mucositis ($n = 8$), neutropenia ($n = 4$) and thrombocytopenia Thrombocytopenia was only observed in patients in cohort three (two patients grade I, one grade II) and cohort four (one grade III). There were no grade IV toxicities.

Four patients discontinued treatment due to toxicity (one patient in cohort two after five cycles due to IFNα-induced pyrexia, one patient in cohort three after one cycle with persistent neutropenia on 5-FU and folinic acid alone, two patients in cohort four after four cycles, one with neutropenia and the other with persistent diarrhoea). Two patients required treatment to be delayed for 1 week and two patients had a dose reduction in IFNα due to haematological toxicity. There was no association between toxicity and TP induction for individual patients.

### Response

Nineteen patients were evaluable for response (one discontinued after one cycle due to toxicity and one withdrew from the study after one cycle). There were no complete or partial responses. Six patients (32%) had stable disease (SD) for at least 3 months. These included five patients who were previously untreated with chemotherapy (tumour types: colorectal, pancreas, cholangiocarcinoma, carcinoid and unknown primary adenocarcinoma), and one patient with gastric carcinoma previously treated with mitomycin C and oral etoposide. Thirteen patients had progressive disease (PD). There was no significant difference in TP levels between patients with SD or PD.

### DISCUSSION

Thymidine phosphorylase (TP) is an important enzyme for the metabolic activation of 5-FU. TP can be upregulated by IFNα, although clinical trials adding IFNα to schedules of 5FU and folinic acid have not shown improved tumour responses (Kosmidis *et al*, 1996; Seymour *et al*, 1996). In order to evaluate the effect of IFNα in vivo we have treated patients with advanced carcinomas with a standard schedule of 5-FU and folinic acid and analysed the effect of different doses and schedules of IFNα on TP activity in PBLs as a pharmacodynamic end-point.

 © 2000 Cancer Research Campaign

We have shown that there is considerable heterogeneity in constitutive PBL TP activity in this group of cancer patients and that treatment with 5-FU and folinic acid alone did not increase TP activity. Addition of a single dose of IFNα to this schedule significantly upregulates PBL TP protein activity within 2 h of administration with a 20% or greater increase in activity in most patients (64%) by 24 h, confirming this as one of the metabolic effects of IFNα in vivo. The level of TP induction was less than previously reported (Makower *et al*, 1997) which may reflect the different assays used. Importantly, the data also demonstrates that, in a significant number of patients (22%), TP activity is not increased by IFNα and in some (14%) it decreases. PBL TP activity, following a single dose of IFNα, was upregulated for at least 8 days and this can be sustained for up to 14 days in the majority of patients (62.5%) who show an initial response. Additional doses of IFNα did not significantly increase TP activity or produce tolerance (Figure 3). Similarly, higher doses of IFNα did not lead to a statistically significant greater level of TP induction.

Induction of TP in vivo should enhance 5-FU cytotoxicity as well as potentially enhancing the efficacy of pro-drugs of 5-FU such as capecitabine. In this study many of the patients were heavily pre-treated or had tumours resistant to conventional cytotoxic therapy. There were no partial or complete responses, although 32% of patients had stable disease for at least 3 months after completion of treatment. The addition of IFNα to the treatment was generally well tolerated with no grade IV toxicities. PBL TP levels in individual patients were not significantly associated with tumour response or toxicity. TP is important in tumour angiogenesis as well as 5-FU activation. Induction of TP should therefore be considered with caution because of evidence that elevated levels may be associated with a worse outcome in some tumour types (Yoshikawa *et al*, 1999). Whilst PBL TP activity was measured for ease of sampling, the direct effect of IFNα on tumour cells in vivo is not known. Future studies are needed to correlate TP activity in fine-needle aspirates from accessible cancers with PBL TP activity.

In randomized studies the addition of IFNα, at a dose of 5 and 6 MIU on alternate days, to schedules of 5-FU and folinic acid, does not improve responses. This dose and schedule of IFNα, based on phase I studies, adversely effects the quality of life of patients receiving treatment (Seymour *et al*, 1994*a*; Wadler *et al*, 1990*a*). We attempted to address whether there was biological evidence in vivo for using alternate day schedules of IFNα compared to those with higher and lower doses of interferon administered at different frequencies. In the four cohort groups treated there was no consistent pattern for TP activity between cohorts or across cycles. While the numbers of patients treated were small, there was no suggestion that treating patients with IFNα 9 MIU/m$^2$ three times per week led to enhanced TP induction compared with a single dose of IFNα every 14 days. Equally, the same total dose of interferon administered by different schedules did not significantly affect TP induction.

The heterogeneity in constitutive TP activity and the response of individual patients to IFNα meant that there were no statistically significant differences in the level of TP induction between cohorts. A single dose of IFNα, as low as 3 MIU/m$^2$ before each cycle of 5-FU and folinic acid, led to a rapid and sustained rise in PBL TP activity in some patients, suggesting that this dose may be as biologically active and better tolerated than more intensive schedules. In other patients there was no induction of TP activity with IFNα and in a small number there appeared to be a reduction in activity. The mechanism of action of IFNα in vivo is not fully understood. The effect on other cytokines, as well as anti-angiogenic actions, will also be important in clinical efficacy.

This study indicates that measurement of TP activity in PBLs from patients receiving chemotherapy is possible and that there is considerable heterogeneity in both baseline activity and in response to IFNα. The variability between individuals in their biological response to IFNα may provide an indicator for which patients are likely to benefit from the addition of IFNα to a standard schedule of 5-FU and folinic acid with implications for treatment in terms of toxicity and cost. The use of IFNα at a maximal tolerated dose may not be the most biologically active schedule in vivo with doses as low as 3 MIU/m$^2$ every 2 weeks, leading to rapid and sustained induction of TP. The design of larger studies modulating 5-FU with IFNα should therefore incorporate biological markers such as TP activity as a pharmacodynamic end-point.

### ACKNOWLEDGEMENT

This work was funded by the Imperial Cancer Research Fund.

### REFERENCES

Advanced Colorectal Cancer Meta-Analysis Project (ACCM-AP) (1992) Modulation of fluorouracil by leucovorin in patients with advanced colorectal cancer: evidence in terms of response rate. *J Clin Oncol* **10**: 896–903

Corfu-A Study Group (1995) Phase III randomized study of two fluorouracil combinations with either interferon alfa-2a or leucovorin for advanced colorectal cancer. *J Clin Oncol* **13**: 921–928

De Gramont A, Krulik M, Cady J, Lagadec B, Maisani JE, Loiseau JP, Grange JD, Gonzalez-Canali G, Demuynck B and Louvet C (1988) High-dose folinic acid and 5-fluorouracil bolus and continuous infusion in advanced colorectal cancer. *European Journal of Cancer & Clinical Oncology* **24**: 1499–1503

Fox SB, Moghaddam A, Westwood M, Turley H, Bicknell R, Gatter KC and Harris AL (1995) Platelet-derived endothelial cell growth factor/thymidine phosphorylase expression in normal tissues: an immunohistochemical study. *J Pathol* **176**: 183–190

Fox SB, Westwood M, Moghaddam A, Comley M, Turley H, Whitehouse RM, Bicknell R, Gatter KC and Harris AL (1996) The angiogenic factor platelet-derived endothelial cell growth factor/thymidine phosphorylase is up-regulated in breast cancer epithelium and endothelium. *Br J Cancer* **73**: 275–280

Giatromanolaki A, Koukourakis MI, Comley M, Kaklamanis L, Turley H, O'Byrne K, Harris AL and Gatter KC (1997) Platelet-derived endothelial cell growth factor (thymidine phosphorylase) expression in lung cancer. *J Pathol* **181**: 196–199

Houghton JA, Morton CL, Adkins DA and Rahman A (1993) Locus of the interaction among 5-fluorouracil, leucovorin, and interferon-alpha 2a in colon carcinoma cells. *Cancer Res* **53**: 4243–4250

Kosmidis PA, Tsavaris N, Skarlos D, Theocharis D, Samantas E, Pavlidis N, Briassoulis E and Fountzilas G (1996) Fluorouracil and leucovorin with or without interferon alfa-2b in advanced colorectal cancer: analysis of a prospective randomized phase III trial. Hellenic Cooperative Oncology Group. *J Clin Oncol* **14**: 2682–2687

Makower D, Wadler S, Haynes H and Schwartz EL (1997) Interferon induces thymidine phosphorylase/platelet derived endothelial cell growth factor expression in vivo *Clinical Cancer Research* **3**: 923–929

Matthews JN, Altman DG, Campbell MJ and Royston P (1990) Analysis of serial measurements in medical research. *BMJ* **300**: 230–235

Moghaddam A and Bicknell R (1992) Expression of platelet-derived endothelial cell growth factor in Escherichia coli and confirmation of its thymidine phosphorylase activity. *Biochemistry* **31**: 12141–12146

Moghaddam A, Zhang HT, Fan TP, Hu DE, Lees VC, Turley H, Fox SB, Gatter KC, Harris AL and Bicknell R (1995) Thymidine phosphorylase is angiogenic and promotes tumor growth. *Proc Nat Acad Sci USA* **92**: 998–1002

Moran RG and Scanlon KL (1991) Schedule-dependent enhancement of the cytotoxicity of fluoropyrimidines to human carcinoma cells in the presence of folinic acid. *Cancer Res* **51**: 4618–4623

O'Brien TS, Fox SB, Dickinson AJ, Turley H, Westwood M, Moghaddam A, Gatter KC, Bicknell R and Harris AL (1996) Expression of the angiogenic factor

© 2000 Cancer Research Campaign 

thymidine phosphorylase/platelet-derived endothelial cell growth factor in primary bladder cancers. *Cancer Res* **56**: 4799–4804
Patterson AV, Zhang H, Moghaddam A, Bicknell R, Talbot DC, Stratford IJ and Harris AL (1995) Increased sensitivity to the prodrug 5′-deoxy-5-fluorouridine and modulation of 5-fluoro-2′-deoxyuridine sensitivity in MCF-7 cells transfected with thymidine phosphorylase. *Br J Cancer* **72**: 669–675
Ragnhammar P, Blomgren H, Edler D, Lundell G, Magnusson I and Sonnenfeld T (1995) Different dose regimens of 5-fluorouracil and interferon-alpha in patients with metastatic colorectal carcinoma. *Eur J Cancer* **31A**: 315–320
Schwartz EL, Baptiste N, O'Connor CJ, Wadler S and Otter BA (1994) Potentiation of the antitumor activity of 5-fluorouracil in colon carcinoma cells by the combination of interferon and deoxyribonucleosides results from complementary effects on thymidine phosphorylase. *Cancer Res* **54**: 1472–1478
Schwartz EL, Baptiste N, Wadler S and Makower D (1995) Thymidine phosphorylase mediates the sensitivity of human colon carcinoma cells to 5-fluorouracil. *J Biol Chem* **270**: 19073–19077
Seymour MT, Johnson PW, Hall MR, Wrigley PF and Slevin ML (1994*a*) Double modulation of 5-fluorouracil with interferon alpha 2a and high-dose leucovorin: a phase I and II study. *Br J Cancer* **70**: 719–723
Seymour MT, Patel N, Johnston A, Joel SP and Slevin ML (1994*b*) Lack of effect of interferon alpha 2a upon fluorouracil pharmacokinetics. *Br J Cancer* **70**: 724–728
Seymour MT, Slevin ML, Kerr DJ, Cunningham D, James RD, Ledermann JA, Perren TJ, McAdam WA, Harper PG, Neoptolemos JP, Nicholson M, Duffy AM, Stephens RJ, Stenning SP and Taylor I (1996) Randomized trial assessing the addition of interferon alpha-2a to fluorouracil and leucovorin in advanced colorectal cancer. Colorectal Cancer Working Party of the United Kingdom Medical Research Council. *J Clin Oncol* **14**: 2280–2288
Takahashi Y, Bucana CD, Akagi Y, Liu W, Cleary KR, Mai M and Ellis LM (1998) Significance of platelet-derived endothelial cell growth factor in the angiogenesis of human gastric cancer. *Clinical Cancer Research* **4**: 429–434
Takebayashi Y, Yamada K, Miyadera K, Sumizawa T, Furukawa T, Kinoshita F, Aoki D, Okumura H, Yamada Y, Akiyama S and Aikou T (1996) The activity and expression of thymidine phosphorylase in human solid tumours. *Eur J Cancer* **32A**: 1227–1232
Wadler S, Goldman M, Lyver A and Wiernik PH (1990*a*) Phase I trial of 5-fluorouracil and recombinant alpha 2a-interferon in patients with advanced colorectal carcinoma. *Cancer Res* **50**: 2056–2059
Wadler S, Wersto R, Weinberg V, Thompson D and Schwartz EL (1990*b*) Interaction of fluorouracil and interferon in human colon cancer cell lines: cytotoxic and cytokinetic effects *Cancer Res* **50**: 5735–5739
Wadler S, Lembersky B, Atkins M, Kirkwood J and Petrelli N (1991) Phase II trial of fluorouracil and recombinant interferon alfa-2a in patients with advanced colorectal carcinoma: an Eastern Cooperative Oncology Group study. *J Clin Oncol* **9**: 1806–1810
Wadler S, Horowitz R, Mao X and Schwartz EL (1996) Effect of interferon on 5-fluorouracil-induced perturbations in pools of deoxynucleotide triphosphates and DNA strand breaks. *Cancer Chemother Pharmacol* **38**: 529–5235
Woolmark N, Bryant J, Smith R, Grem J, Allegra C, Hyams D, Atkins J, Dimitrov N, Oishi R, Prager D, Fehrenbacker L, Romond D, Colangelo L and Fisher B (1998) Adjuvant 5-fluorouracil and leucovorin with or without interferon alfa-2a in colon carcinoma: National Surgical Adjuvant Breast and Bowel Project protocol C-05 *J Natl Cancer Inst* **90**: 1810–1816
Yoshikawa T, Suzuki K, Kobayashi O, Sairenji M, Motohashi H, Tsuburaya A, Nakamura Y, Shimizu A, Yanoma S and Noguchi Y (1999) Thymidine phosphorylase/platelet-derived endothelial cell growth factor is upregulated in advanced solid types of gastric cancer *Br J Cancer* **79**: 1145–1150

 *© 2000 Cancer Research Campaign*
